# Work-style Reform: A Deeper Look at Autonomy

**DOI:** 10.31662/jmaj.2025-0275

**Published:** 2025-08-08

**Authors:** Shigeki Matsubara

**Affiliations:** 1 Department of Obstetrics and Gynecology, Jichi Medical University, Tochigi, Japan; 2 Department of Obstetrics and Gynecology, Koga Red Cross Hospital, Koga, Ibaraki, Japan; 3 Medical Examination Center, Ibaraki Western Medical Center, Chikusei, Ibaraki, Japan

**Keywords:** burnout, motivation, residency, resident, work-style reform

## Dear Editors,

Nakao et al. ^(1)^ showed: after the “work-style reform,” pediatric residents’ working hours and burnout scores decreased. Another viewpoint may complement the discussion.

The aims of work-style reform are multiple. The reform is for both residents and society, including patients. Hard work sometimes causes burnout and even suicide. Doctors should have enough rest and time for their personal lives. Also, fatigue due to overwork can cause medical errors, which harm patients and cause social loss. Here, I confine the discussion to the former. Does a “shorter hospital stay” really serve residents’ interests? My answer is: while a “forced and meaningless” longer stay does not benefit residents, an “autonomous and meaningful” longer stay does.

Recently, burnout has often been explained in the context of Self-Determination Theory (SDT) ^(2), (3)^, which involves three psychological aspects―autonomy, competence, and relatedness. Autonomy means voluntary choice in professional activities. Competence represents the personal desire to engage with challenging tasks. Relatedness means connection with team members, cultivating professional relationships. Individuals possessing or obtaining these three tend to be highly motivated and actively participate in activities they find meaningful. This fits even a non-psychology-specialist like me.

I believe future studies should be directed toward the following:

1. Do residents find their individual institute’s system satisfactory in terms of autonomy, competence, and relatedness?

2. Do program leaders consider the education system from the perspective of these three elements?

3. If the answer to 1 and/or 2 is “no,” what should be changed?

Work hours were reduced through the “Work short!” approach ^(1)^: this observation is paramount. However, future studies should move beyond merely “reducing negatives” like burnout. We should ask whether the reform actively “increases positives”―namely, residents’ satisfaction and motivation, in terms of SDT. In short, does this reform make residents “happy”―happy as professionals, now, at the completion of residency, and throughout their careers?

I previously stated that residents’ individual wishes for a longer hospital stay should be respected ^(4)^. I have now widened the view. A longer stay is welcome only if residents’ personal desire to “learn more” is met. In doing so, program leaders should consider the three elements: autonomy, competence, and relatedness. This differs from resident to resident.

Writing is “hard work” for me; however, writing is unlikely to push me toward burnout: I possess full autonomy, competence, and relatedness. One willingly moves only when motivated. Uniformly prohibiting long stays may not be a blessing, but a kind of torture for some residents.

## Article Information

### Conflicts of Interest

None

### Acknowledgement

The author thanks doctors Yuji Takei (Department of Obstetrics and Gynecology, Jichi Medical University, Japan) and Daisuke Matsubara (Division of Community Medicine and Department of Pediatrics, Jichi Medical University, Japan) for their help.

### Author Contributions

Shigeki Matsubara: Identification of the significance and manuscript writing.

### Approval by Institutional Review Board (IRB)

Not applicable

### Data Availability

Data sharing does not apply to this article, as no new data were created or analyzed in this study.

### Patient anonymity

Not applicable.

### Informed consent

Not applicable.
